# Safety, tolerability and efficacy of the glutaminyl cyclase inhibitor PQ912 in Alzheimer’s disease: results of a randomized, double-blind, placebo-controlled phase 2a study

**DOI:** 10.1186/s13195-018-0431-6

**Published:** 2018-10-12

**Authors:** Philip Scheltens, Merja Hallikainen, Timo Grimmer, Thomas Duning, Alida A Gouw, Charlotte E Teunissen, Alle Meije Wink, Paul Maruff, John Harrison, Caroline M van Baal, Suzanne Bruins, Inge Lues, Niels D Prins

**Affiliations:** 1 Alzheimer Center and Department of Neurology, Amsterdam Neuroscience, VU University Medical Center, Amsterdam, The Netherlands; 2 University of Eastern Finland, Institute of Clinical Medicine, Kuopio, Finland; 3 Department of Psychiatry and Psychotherapy, Klinikum rechts der Isar, Technische Universität München, Munich, Germany; 4 Department of Neurology, University of Münster, Münster, Germany; 5 Department of Clinical Neurophysiology and MEG Center, Amsterdam Neuroscience, VU University Medical Center, Amsterdam, The Netherlands; 6 Department of Clinical Chemistry, Neurochemistry Laboratory and Biobank, Amsterdam Neuroscience, VU University Medical Center, Amsterdam, The Netherlands; 7 Department of Radiology, Nuclear Medicine and PET Research, Amsterdam Neuroscience, VU University Medical Center, Amsterdam, The Netherlands; 8 Cogstate Ltd, Melbourne, Australia; 9 Institute of Psychiatry, Psychology and Neuroscience, King’s College London, London, UK; 10 Julius Center for Health Sciences and Primary Care, UMC Utrecht, Utrecht, The Netherlands; 11 Probiodrug AG, Halle, Germany; 12 Brain Research Center, Amsterdam, The Netherlands

**Keywords:** Alzheimer’s disease, Glutaminyl cyclase inhibitor, PQ912, Phase 2a

## Abstract

**Background:**

PQ912 is an inhibitor of the glutaminyl cyclase enzyme that plays a central role in the formation of synaptotoxic pyroglutamate-A-beta oligomers. We report on the first clinical study with PQ912 in subjects with biomarker-proven Alzheimer’s disease (AD). The aim was to determine the maximal tolerated dose, target occupancy and treatment-related pharmacodynamic effects. The exploratory efficacy readouts selected were tailored to the patient population with early AD. The therapeutic approach focuses on synaptic dysfunction as captured by various measures such as electroencephalography (EEG), synaptic biomarkers and sensitive cognitive tests.

**Methods:**

This was a randomized, double-blind, placebo-controlled trial evaluating the safety, tolerability and efficacy of PQ912 800 mg twice daily (bid) for 12 weeks in subjects with mild cognitive impairment or mild dementia due to AD. The 120 enrolled subjects were treatment-naïve at the start of the study, had confirmed AD biomarkers in their cerebrospinal fluid at screening and had a Mini Mental State Examination score between 21 and 30. After 1 week of treatment with 400 mg bid, patients were up-titrated to 800 mg bid for 11 weeks. Patients were randomized 1:1 to either PQ912 or placebo. The primary composite endpoints were to assess safety and tolerability based on the number of patients who discontinued due to (serious) adverse events (safety), and based on dose adjustment during the treatment period and/or nonadherence to randomized treatment (tolerability). All randomized subjects who took at least one dose of the study treatment or placebo were used for safety analyses.

**Results:**

There was no significant difference between treatments in the number of subjects with (serious) adverse events, although there were slightly more patients with a serious adverse event in the PQ912 group compared to placebo. More subjects treated with PQ912 discontinued treatment due to adverse events, mostly related to gastrointestinal and skin/subcutaneous tissue disorders. PQ912 treatment resulted in a significant reduction in glutaminyl cyclase activity, which resulted in an average target occupancy of > 90%. A significant reduction of theta power in the EEG frequency analysis and a significant improvement in the One Back test of our Neuropsychological Test Battery was observed. The exploratory biomarker readouts, neurogranin for synaptic toxicity and YKL-40 as a marker of inflammation, appear to be sensitive enough to serve as efficacy markers in the next phase 2b study.

**Conclusions:**

The maximal tolerated dose of PQ912 has been identified and the results support future studies at still lower doses reaching > 50% target occupancy, a longer up-titration phase to potentially induce adaptation and longer treatment periods to confirm the early signals of efficacy as seen in this study.

**Trial registration:**

Clinicaltrials.gov, NCT 02389413. Registered on 17 March 2015.

**Electronic supplementary material:**

The online version of this article (10.1186/s13195-018-0431-6) contains supplementary material, which is available to authorized users.

## Background

**Fig. 1:**
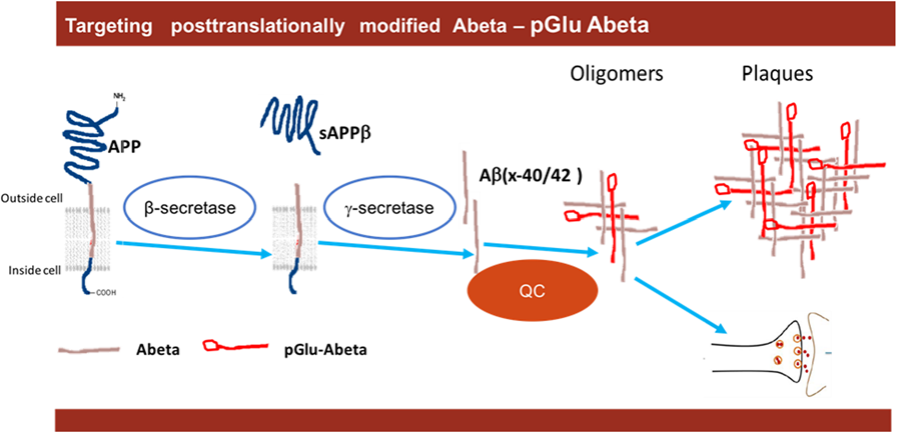
Schematic drawing of QC inhibitor approach. pGlu-Aβ is posttranslationally formed by QC from N-terminally truncated Aβ versions carrying a glutamate at position N3 and N11. pGlu-Aβ seeds Aβ oligomers which are highly synaptotoxic and neurotoxic. Aβ amyloid beta, pGlu pyroglutamate, QC glutaminyl cyclase, sAPPβ soluble amyloid precursor protein beta

Alzheimer’s disease (AD) is the most prevalent form of dementia, affecting more than 40 million people worldwide with prevalence expected to double by 2030 [1]. At present, only symptomatic treatment is available, and there is a strong need for disease-modifying treatments. Although the etiology of AD is not understood completely, there is agreement that beta-amyloid (Aβ) plaques and tau tangles are central to its biology [2–5]. Different Aβ species can form soluble oligomers of varying size. However, in contrast to insoluble Aβ plaques, soluble Aβ oligomers are accepted as the primary source of toxicity because they lead to synaptic impairment, reduce spike numbers, impair neuronal connectivity and result in tau-dependent neurodegeneration over the course of the disease [6, 7]. Consequently, Aβ oligomers may be an important therapeutic target for halting AD. Studies into the molecular events of Aβ oligomer composition and formation show the importance of the enzyme glutaminyl cyclase (QC) in these processes [8]. Increasing full-length Aβ_1–40_ or Aβ_1–42_ levels early in AD leads to activation of various peptidases (e.g., neuroendopeptidase), which in turn cause truncated versions of Aβ with an N-terminal glutamate at positions 3 and 11. These truncated versions are then modified by glutamate cyclization to pyroglutamate (pGlu) by QC, as illustrated in Fig. 1. The pGlu-Aβ-seeded Aβ oligomers possess a very high synaptotoxic, neurotoxic and proinflammatory potential in in-vitro and animal models [8, 9]. pGlu-Aβ-seeded oligomers strongly suppressed LTP in brain slices, an indicator of synaptic impairment [8]. In a translational study in an AD animal model, PQ912 significantly rescued impaired spatial learning and memory at a dose which achieved between 50 and 70% QC inhibition in the spinal fluid [9]. pGlu-Aβ has been shown to induce changes in the secondary and tertiary structure of oligomers and these changes most likely are responsible for the increased synaptotoxic propensity of pGlu-Aβ-containing aggregates [8, 10, 11]. The mechanisms of synaptotoxicity of pGlu-Aβ oligomers have been shown to be different compared to Aβ oligomers [12].

The discovery of pGlu-Aβ pathobiology provides a foundation for a new therapeutic approach in AD where inhibition of the activity of QC could reduce the production of pGlu-Aβ in the brain. This could subsequently alleviate the acute and chronic neurotoxic effects of pGlu-Aβ oligomers. Consequently, long-term inhibition of QC activity would allow synaptic regeneration and reduced neuronal death rates which should manifest clinically as a reduction in cognitive decline and lower rates of clinical disease progression. PQ912, an inhibitor of QC, has undergone rigorous preclinical and clinical investigations including in-vitro and in-vivo animal and phase 1 studies [9, 13] and is a first-in-class QC inhibitor in clinical development. This phase 2a study had the objective to investigate the highest PQ912 dose that was used in an earlier phase 1 study of the same compound [13], to evaluate safety and tolerability signals in treatment-naïve subjects with mild cognitive impairment (MCI) due to AD or mild dementia due to AD after 12 weeks of treatment and also to evaluate the usefulness of various efficacy measurements for future studies using longer treatment durations.

## Methods

### Study design

This was a multicenter, randomized, double-blind, placebo-controlled, parallel-group safety and tolerability phase 2 study of PQ912. Efficacy was assessed in an exploratory manner.

Subjects satisfying all selection criteria at screening were randomly assigned in a 1:1 ratio to receive either PQ912 or placebo for 12 weeks. Dosage of PQ912 was 400 mg bid in the first week, followed by 800 mg bid for 11 weeks. During the study, visits to the study site occurred at baseline, after 3, 6 and 12 weeks on treatment and 28 days after the end of treatment.

Procedures at screening included documentation of medical history, physical and neurological examination, assessments of vital signs, ECG, EEG, MMSE, Geriatric Depression Scale, Neuropsychological Test Battery and MRI including resting state functional (RSf)MRI. Blood and urine samples were collected for blood chemistry, hematology, parameters related to QC substrates (thyroid-stimulating hormone (TSH), total triiodothyronine (T3), free prohormone thyroxine (T4), testosterone) and for ApoE genotyping. A CSF sample was collected for biomarker assessment.

The MMSE and Neuropsychological Test Battery were repeated at baseline and end of treatment (EOT); CSF sampling, MRI, ECG and EEG were repeated at EOT only. Physical and neurological examination, vital signs and blood and urine sampling were repeated at each study visit. (S)AEs and concomitant medication were reported throughout the study.

At baseline and EOT, a 20-min resting-state EEG was recorded against a common reference at 21 electrode positions of the 10–20 system. Patients were seated in a slightly reclined chair in a sound-attenuated but fully lit room and were instructed to keep their eyes closed and stay awake. EEG technicians monitored the recording carefully and alerted the patients by sound stimuli at the first signs of drowsiness.

Central analysis/reading was done for clinical laboratory, genotyping, MRI images, EEG data and biomarkers. Cogstate performed central monitoring on the computerized test data.

### Subjects

A total of 120 treatment-naïve men (*n* = 56) and women (*n* = 64), aged 51–85 years, with a diagnosis of mild cognitive impairment due to AD or mild dementia due to AD, according to the Alzheimer Association–National Institute on Aging criteria [14, 15], were included in the study. Other inclusion criteria were a Mini Mental State Examination (MMSE) score between 21 and 30, and a brain magnetic resonance imaging (MRI) scan consistent with the diagnosis of the disease. In addition, subjects had to show a positive AD biomarker signature at screening.

### Safety and tolerability endpoints

Two primary safety and tolerability composite endpoints were defined. Safety was based on: discontinuation of subject due to a serious adverse event (SAE); or discontinuation of subject due to an adverse event (AE) with severity ≥ 3 according to the Common Terminology Criteria for Adverse Events (CTCAE); or discontinuation of subject due to an extreme laboratory parameter. Tolerability was based on: dose adjustment during the treatment period; and/or nonadherence to randomized treatment.

Secondary tolerability endpoints were: number of SAEs and AEs with severity ≥ 3 according to the CTCAE; the time to first appearance of AEs; time to dose adjustment; time to nonadherence; and a composite of time to dose adjustment, nonadherence, discontinuation due to SAE, AE with severity ≥ 3 according to the CTCAE or extreme laboratory parameters.

Other safety measures were AEs, vital signs, ECG measurements, clinical laboratory tests, changes on brain MRI scans, and physical and neurologic examinations.

### Efficacy endpoints

The following endpoints were defined for exploratory efficacy.

- CSF biomarkers: diagnostic biomarkers Aβ_1–42_, tau and P-tau (Innotest, Fujirebio); and exploratory biomarkers QC activity, primary target of inhibitor (Evotec AG), neurogranin and beta secretase I (ADxNeurosciences/Euroimmun), Contactin 2 (R and D systems, duoset), neurofilament light chain (Uman diagnostics) and Chitinase-3-like protein 1 (CHI3L1 = YKL-40) (Quidel Corporation).
- Neuronal oscillatory activity and network anaysis as measured by EEG: mean peak frequency in the parieto-occipital region, global relative alpha and theta power (8–13 Hz and 4–8 Hz, respectively), mean global phase lag index in the alpha band and network topology measures in the alpha band, based on the minimum spanning tree of the full network—mean phase lag index, leaf fraction and tree hierarchy in the alpha band.
- MRI: normalized brain volume at screening and percentage brain volume change at EOT.
- RSfMRI: mean *z*-statistic default mode network (DMN), mean eigenvector centrality values and mean path length and clustering coefficient.
- Cognition: episodic memory (average of standardized scores of the OCL Test, ISLT and ISLT-delayed recall), executive function (average of standardized scores of LFT, CFT and One Back Test), attention (average of standardized scores of Detection Test and Identification Test), overall cognition (average of standardized scores of all cognitive measures; scores from at least six tests needed for calculation) and MMSE.

### QC activity and inhibition

QC activity in CSF was measured by Evotec AG (Hamburg) and PQ912 by Swiss Bioquant (Basel) as described by Lues et al. [13].

In-vivo QC target occupancy (TO) was calculated from PQ912 CSF levels for CSF samples collected within 24 h after the last compound intake at the EOT visit by applying the following formula:

TO (%) = 100 × *C* / (*K*_i_ + *C*)

where TO = target occupancy (%), *K*_i_ = inhibitory constant of PQ912 (25 nM) and *C* = measured CSF concentration of PQ912 Additional file 2: Table S1.

### Statistical analysis

No formal sample size calculation was conducted, since this was not possible in the absence of any clinically relevant incidence of adverse events or drug discontinuations in a previously conducted comprehensive phase 1 study for PQ912. In order to gain sufficient safety and tolerance information, we planned a sample size of 110 subjects (55 per group) which allows an exposure of 10 patient-years in each study arm (55 subjects × 3 months minus potential early discontinuation) in this first in-patient study.

The effect of treatment on the primary composite endpoints was evaluated using Fisher’s exact tests (α_(2-sided)_ = 0.05). The relative risk (RR) and risk difference (RD) were reported with a 95% confidence interval (CI). Kaplan–Meier plots and log-rank tests were used for time to event outcomes (first appearance of AEs, dose adjustment, nonadherence or a composite of dose adjustment, nonadherence, discontinuation due to SAE and AE with severity ≥ 3 according to CTCAE or extreme laboratory parameters). Descriptive statistics were used for other safety measures.

Analysis of the exploratory efficacy parameters was performed in three distinct populations: intention to treat (ITT), defined as all randomized subjects who took at least one dose of the study treatment or placebo; modified ITT (mITT), defined as the ITT population excluding subjects who started using acetylcholinesterase inhibitor during treatment period; and per protocol (PP), defined as all randomized subjects without major protocol deviations. Analysis of the cerebrospinal fluid (CSF) biomarker endpoints was conducted in the PP population because in this population the timing of last treatment and sampling occurred within a 24-h window. Efficacy endpoints were first tested for normality, and transformed if necessary. All CSF biomarkers were log_10_ transformed before statistical analysis, as well as two EEG parameters and one RSfMRI parameter. Single imputation was performed for ITT and mITT analyses. α_(2-sided)_ = 0.05 was used without adjustment for multiple comparisons.

An ANCOVA with treatment, gender, ApoE and country (stratification) as factors and time between screening and baseline, baseline measure of the endpoint and age as covariates was performed for cognitive, CSF and neuronal network endpoints. Cohen’s *D* (*d*) treatment effect sizes and differences between treatment groups, corrected for the previously mentioned covariates (δ, with 95% CI), were reported for these parameters. Due to many observations below the limit of quantification, data for pGlu-Aβ monomer and pGlu-Aβ oligomer were dichotomized (yes or no quantifiable) and analyzed by Mantel–Haenszel chi-square statistics.

## Results

### Demographics

**Fig. 2:**
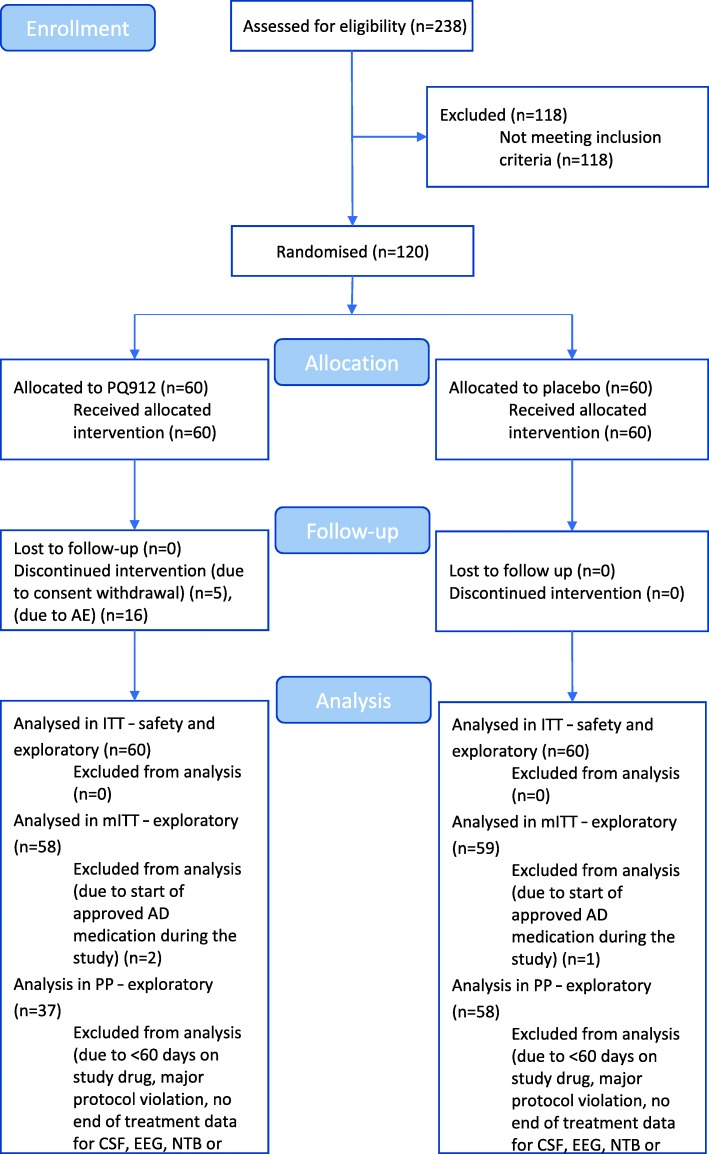
CONSORT flow diagram. AD Alzheimer’s disease, AE adverse event, CSF cerebrospinal fluid, EEG electroencephalography, ITT intention to treat, mITT modified intention to treat, MRI magnetic resonance imaging, NTB Neuropsychological Test Battery, PP per protocol

Out of a total of 238 subjects who were screened, 120 subjects were enrolled in the study and randomized to treatment with PQ912 (*n* = 60) or placebo (*n* = 60); 115 subjects completed the end of treatment (EOT) visit (55 in the PQ912 group and 60 in the placebo group). These numbers are also shown in the CONSORT flow diagram (Fig. 2). Five subjects treated with PQ912 withdrew their consent and did not complete the EOT visit. The subject disposition for each study group is presented in Table 1. The only difference between groups at baseline was that the PQ912 group contained more women (60%) than the placebo group (47%). Carriage of the apolipoprotein E (APoE) E4 allele was as expected and equivalent in both groups (63% in the PQ912 group and 72% in the placebo group).

**Table 1:** Demographics and other baseline characteristics

	Statistic	Placebo (*N* = 60)	PQ912 (*N* = 60)
Age (years)	Mean (SD)	72 (7)	70.8 (8)
Sex			
Female	*n* (%)	28 (47)	36 (60)
Male	*n* (%)	32 (53)	24 (40)
MMSE	Mean (SD)	24.8 (3)	25.2 (3)
APoE			
E4	*n* (%)	43 (72)	38 (63)

*APoE* apolipoprotein E, *MMSE* Mini Mental State Examination, *N* number of subjects in population, *n* number of subjects, *SD* standard deviation

### Safety and tolerability

There were slightly more patients with a serious adverse event (SAE) in the PQ912 group compared to placebo, although statistically not significant. In the PQ912 group, eight subjects reported 13 SAEs and in the placebo group, five subjects reported five SAEs. The SAEs reported were heterogeneous in nature and showed no pattern pointing to a specific effect of PQ912. Six subjects in the PQ912 group discontinued treatment due to the SAEs and none in the placebo group.

The number of subjects with treatment emergent adverse events (TEAEs) was not different between study groups, although the PQ912-treated subjects reported slightly more TEAEs. In the PQ912 group, 45 subjects reported 135 TEAEs, while 40 subjects in the placebo group reported 103 TEAEs. The majority of AEs were classified as mild or moderate in severity. Subjects in the PQ912 group reported AEs most frequently in the system organ class (SOC) categories of gastrointestinal disorders (*n* = 21), infections and infestations (*n* = 17), and skin and subcutaneous tissue disorders (*n* = 15). The most frequently reported TEAEs in the placebo group were in the SOC categories of gastrointestinal disorders (*n* = 12) and infections and infestations (*n* = 17). Five placebo subjects reported skin and subcutaneous tissue disorders (Table 2).

**Table 2:** Treatment emergent adverse events summary and distribution by Medical Dictionary for Regulatory Activities (MedDRA) system organ classes (SOC) (with ≥ 3 subjects reporting in total)

TEAE summary	Placebo (*N* = 60)	PQ912 (*N* = 60)
Subjects with any TEAE	40 (67)	45 (75)
Subjects who discontinued due to TEAE^a^	0	20 (33)
Subjects with TEAE by severity		
Mild	27 (45)	17 (28)
Moderate	12 (20)	20 (33)
Severe	1 (2)	8 (13)
Number of TEAEs	103	135
Subjects with serious TEAEs	3 (5)	8 (13)
Number of SAEs	5	13
Number of serious TEAEs	3	13
Deaths	0	0
MedDRA SOC—preferred term		
Blood and lymphatic system disorders	1	2
Cardiac disorders	2	2
Gastrointestinal disorders	12	21
Abdominal pain upper	3	1
Constipation	0	3
Diarrhea	1	5
Dyspepsia	2	1
Nausea	4	8
Vomiting	1	2
General disorders and administration site conditions	6	5
Hepatobiliary disorders	0	3
Infections and infestations	17	17
Injury, poisoning and procedural complications	5	6
Investigations	5	8
Metabolism and nutrition disorders	2	7
Musculoskeletal and connective tissue disorders	8	5
Nervous system disorders	9	7
Psychiatric disorders	3	4
Renal and urinary disorders	1	3
Skin and subcutaneous tissue disorders	5	15
Rash	2	4
Rash generalized	0	1
Rash maculopapular	1	1
Urticaria	0	4
Vascular disorders	2	3

Data presented as *n* (%) or *n*

*N* number of subjects in population, *n* number of subjects, *SAE* serious adverse event, *SOC* system organ class, *TEAE* treatment emergent adverse event

^a^These subjects may still have completed end-of-study assessment

**Fig. 3:**
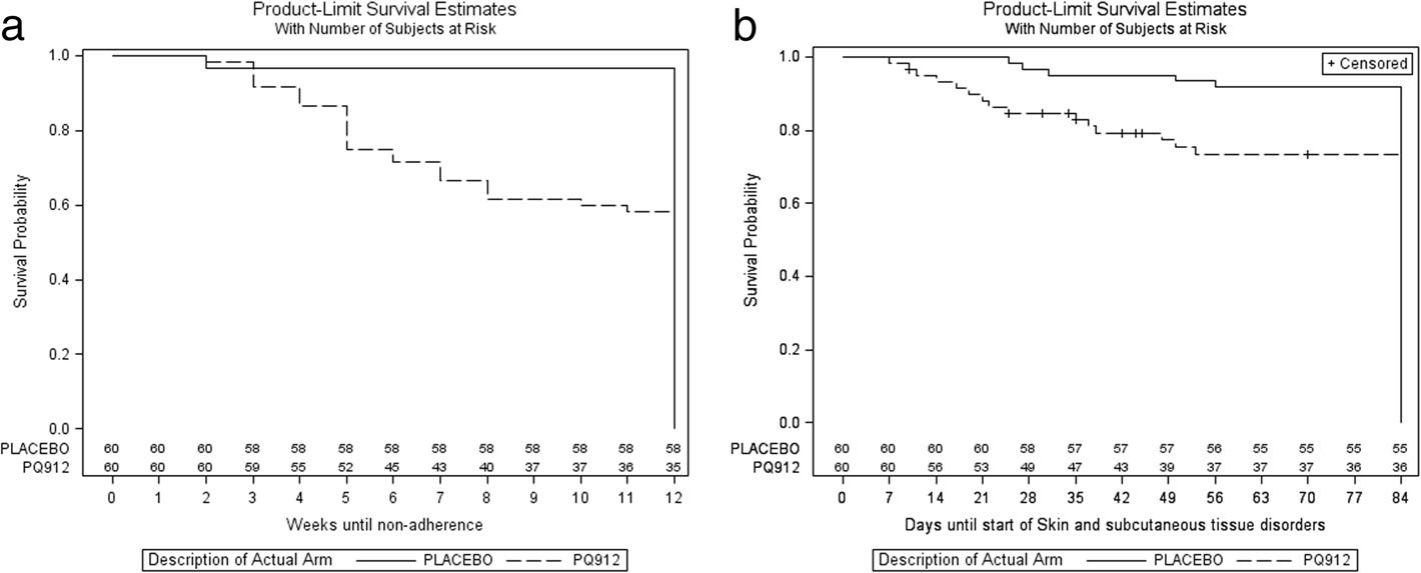
**a** Time to nonadherence. Intent to treat population. **b** Time to start of TEAEs of skin and subcutaneous tissues disorders . Intent to treat population

The greatest differences between incidences of TEAEs were observed in the SOC categories of gastrointestinal disorders and skin and subcutaneous tissue disorders. Within these two categories, subjects in the PQ912 group reported mostly nausea, diarrhea and constipation, and rash and urticaria. Subjects in the placebo group reported mostly nausea, abdominal pain upper and rash in these categories. AEs in the SOC category of infections and infestations were mostly viral upper respiratory tract infection and upper respiratory tract infection, and showed no difference between treatment arms (Table 2). The onset of TEAEs in the SOC of skin and subcutaneous tissue disorders occurred mostly in week 2 to week 8 (Fig. 3a).

In both study groups, a reduction in the prescribed dose from 800 mg to 400 mg twice daily (bid) was observed in five subjects. In the PQ912 group, 26 subjects (43.3%) met the criteria for nonadherence to study treatment; 20 of them discontinued due to AEs, five continued treatment and one withdrew consent. Four of the 20 subjects who discontinued treatment also withdrew consent and did not complete the study. In the placebo group, two subjects (3.3%) qualified for nonadherence to study treatment, but did not discontinue the study. Time to nonadherence was reached significantly earlier for subjects in the PQ912 group compared to placebo (*P* < 0.001). The majority of treatment discontinuations were observed between treatment weeks 2 and 8 (Fig. 3b).

Prospectively defined composite endpoints for safety and for tolerability were significantly different between the PQ912 and placebo groups (*P* = 0.027 for safety and *P* < 0.001 for tolerability, Table 3).

**Table 3:** Safety and tolerability endpoints

	Placebo (*N* = 60), *n* (%)	PQ912 (*N* = 60), *n* (%)	*P*
Safety			
Composite safety	0 (0.0)	6 (10.0)	0.027
Discontinuation of subject due to SAE	0 (0.0)	6 (10.0)	0.027
Discontinuation of subject due to AE with severity ≥ 3 according to CTCAE	0 (0.0)	6 (10.0)	0.027
Discontinuation of subject due to extreme safety laboratory parameters	0 (0.0)	0 (0.0)	–
Number of subjects with SAEs	5 (8.3)^d^	8 (13.3)	0.558
Number of subjects with AEs with severity ≥ 3 according to CTCAE	3 (5.0)^e^	8 (13.3)	0.204
Tolerability			
Composite tolerability	6 (10.0)	27 (45.0)	<.001
Dose adjustment during treatment period^a^	5 (8.3)	5 (8.3)	1.000
Nonadherence to randomized treatment^b^	2 (3.3)	26 (43.3)	<.001
Discontinued due to related AE^c^	0 (0.0)	19 (31.7)	<.001
Discontinued due to nonrelated AE	0 (0.0)	1 (1.7)	1.000
Withdrawal of consent by subject	0 (0.0)	1 (1.7)	1.000
Continued	2 (3.3)	5 (8.3)	0.439

*CTCAE* common terminology criteria for adverse events, *N* number of subjects in population, *n* number of subjects, (*S)AE* (serious) adverse event

^a^Dose adjustment defined as reduction of dose from 800 mg bid to 400 mg bid, as allowed within the study protocol

^b^Nonadherence defined as: using < 75% of prescribed dose in 4 consecutive weeks including at least 1 week with less than 50%; or 3 or more consecutive days in total or 7 days of interrupted use during the full 12 weeks. Subjects could qualify for more than one criterion, but were listed once; reason ranked for relevance for tolerance

^c^Includes possibly related, probably related and related according to investigator

^d^Treatment emergent in three subjects

^e^Treatment emergent in one subject

Overall, we found no clinically relevant differences between the PQ912 and placebo groups in changes over time in any of the clinical chemistry and hematology parameters or parameters related to physiological QC substrates. We observed shifts from normal to out of normal laboratory range values more frequently in the PQ912 group for hemoglobin, hematocrit and alkaline phosphatase, although these values normalized toward EOT and became normal by the follow-up assessment. One subject showed a clinically significant increase in transaminases at EOT, but this did not meet Hy’s law criteria and values normalized after discontinuation of treatment with PQ912. As thyrotropin-releasing hormone (TRH) and gonadotropin-releasing hormone (GnRH) are substrates of QC, the downstream hormones thyroid-stimulating hormone (TSH), T3, T4 and testosterone were determined in blood and were not different between the PQ912 and placebo groups.

PQ912 treatment had no noticeable effect on vital signs and ECG, and no abnormal findings were noted in physical and neurological examinations and in brain magnetic resonance imaging (MRI) scans.

### Efficacy

In total, 120 subjects were randomized to PQ912 treatment or placebo (ITT/mITT/PP populations: *N*_PQ912_ = 60/58/37, *N*_placebo_ = 60/59/58). All measures were prospectively defined in the SAP which was finalized before database lock. Additional file 1 summarizes the mean values and standard deviations of the efficacy measures at baseline and end of treatment and for the changes between end of treatment and baseline with 95% confidence intervals. The effect sizes as expressed by Cohen’s *D* for those measures especially relevant to the concept are separately presented in Table 4.

**Table 4:** Cohen’s *d* effect size for significant efficacy parameters

Domain	Parameter	ITT population	mITT population	PP population	Effect size^a^
CSF	QC activity	1.25***	1.28***	1.66***	↓ large
EEG	Relative theta power	0.29**	0.32***	0.37**	↓ small–moderate
NTB CSF	One Back Test	0.23*	0.23^†^	0.20	↓ small
	Neurogranin	0.16^†^	0.20*	0.12	↓ small
	YKL-40	0.16^†^	0.16^†^	0.20*	↓ small

*CSF* cerebrospinal fluid, *EEG* electroencephalogram, *ITT* intent to treat, *mITT* modified ITT, *NTB* Neuropsychological Test Battery, *PP* per protocol, *QC* glutaminyl cyclase

^a^Arrow indicates direction of treatment effect (↓ decrease)

^†^0.05 < *p* ≤ 0.10

*0.01 < *p* ≤ 0.05

**0.001 < *p* ≤ 0.01

***p ≤ 0.001

#### CSF biomarkers

##### QC activity

**Fig. 4:**
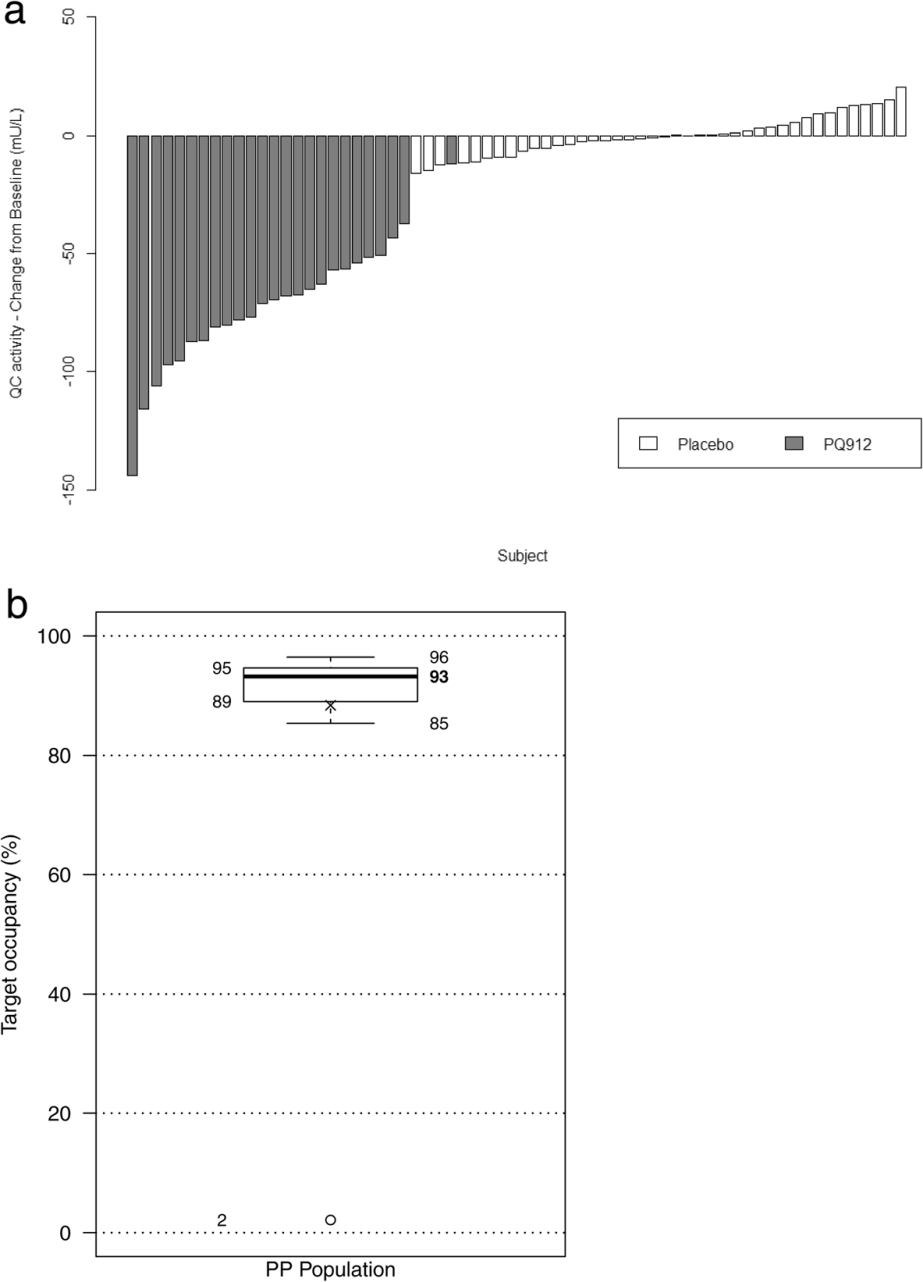
**a** Glutaminyl cyclase activity (mU/L): change from baseline at end of treatment. Per protocol population. **b** In-vivo target occupancy calculated from PQ912 levels. PQ912 treated, per protocol (PP) population

The mean QC activity was 118.5 mU/L (SD 33.1, *n* = 26) at baseline and decreased to 46.0 mU/L (SD 24.3, *n* = 26) at EOT (*P* < 0.001, *d* = 1.66, PP population). In the placebo group, the QC activity levels remained constant (mean level at baseline 121.5 mU/L, SD 31.5; at EOT 121.4 mU/L, SD 32.8, *n* = 41) (Fig. 4a).

With the exception of one outlier with a low target occupancy (TO) of 2.1%, the estimated median TO based on calculation from measured levels of PQ912 in CSF was 93.2% (interquartile range 89.0–94.6%) (Fig. 4b).

##### YKL-40

The effect of PQ912 on change in the level of YKL-40 (a marker of glia activation) from baseline to EOT was statistically significant in the PP population (*P* = 0.025, *d* = 0.20), indicating a stronger decrease in the PQ912 group compared to placebo. The mean of 344.2 pg/ml (SD 150.3) at baseline decreased to 324.3 pg/ml (SD 125.8) at EOT in the PQ912 group (*n* = 26), while in the placebo group there was no change: the mean was 386.5 pg/ml (SD 127.5) at baseline and 381.6 pg/ml (SD 142.9) at EOT (*n* = 41). The decrease of the means was 20 pg/ml (6%) in the PQ912 group and 5 pg/ml (1%) in the placebo group.

##### Neurogranin

While neurogranin levels decreased in the PQ912 group this decrease was not consistently statistically significant different between study groups. In the mITT population there was a small, significant effect (*P* = 0.036, *d* = 0.20), while in the PP and ITT populations changes in mean neurogranin levels were not significantly different, although there was a trend to decrease in the treatment group. In the PP population, the mean of 451.0 pg/ml (SD 193.4) at baseline decreased to 431.8 pg/ml (SD 191.9) at EOT in the PQ912 group (*n* = 26), while in the placebo group the mean was 477.6 (SD 188.1) pg/ml at baseline and 468.4 pg/ml (SD 192.4) at EOT (*n* = 41). The decrease of the means was 19 pg/ml (4%) in the treatment arm and 10 pg/ml (2%) in the placebo group. ApoE had a statistically significant effect on neurogranin (*P* = 0.011, *P* = 0.008 and *P* = 0.025 for ITT, mITT and PP populations, respectively), showing higher neurogranin values for E4 carriers in both study groups.

Diagnostic (Aβ_1–42_, tau or P-tau levels) and other biomarkers (neurofilament light chain, beta secretase-I and Contactin-2) in the CSF did not change significantly across the 12-week period for both study groups.

#### Neuronal oscillatory activity and functional network endpoints

Spectral analyses of the oscillatory electroencephalography (EEG) activity showed a difference in mean change in global relative theta power (4–8 Hz) in the PQ912 group compared to placebo in the ITT (*P* = 0.002, *d* = 0.29), mITT (*P* < 0.001, *d* = 0.32) and PP (*P* = 0.002, *d* = 0.37) populations. In the placebo group, the baseline global relative theta power increased from mean 0.1647 (SD 0.0794) to 0.1762 (SD 0.0819) at EOT (ITT population), whereas it decreased slightly in the PQ912 group from mean 0.1763 (SD 0.0976) to 0.1695 (SD 0.0881) at EOT (ITT population).

Global functional connectivity, as measured with the phase lag index [16] in the alpha band, and measures reflecting functional network topology (leaf fraction, mean phase lag index and tree hierarchy of the network, represented by its minimum spanning tree, an unbiased subnetwork of the full functional network [17, 18]) did not show differences between treatment groups. Alpha band leaf fraction and tree hierarchy were influenced significantly by the ApoE4 allele (higher values in E4 carriers for alpha band leaf fraction, lower values in E4 carriers for tree hierarchy) with *P* = 0.009 for alpha band leaf fraction and *P* = 0.001 for tree hierarchy, respectively, in each of the ITT, mITT and PP populations.

PQ912 treatment had no effect on brain volume. Analyses of resting state functional (RSf)MRI endpoints showed no PQ912 treatment effects except for a statistically significant effect with lower EOT values in the PQ912 group compared to the placebo group observed in the mean eigenvector centrality of the cuneus and lateral occipital regions (posterior) in the ITT (*P* = 0.005, *d* = 0.58), mITT (*P* = 0.003, *d* = 0.60) and PP (*P* = 0.132 and *d* = 0.39) populations.

#### Cognitive endpoints

With regard to the individual cognitive test scores, we observed a PQ912 treatment effect on the One Back test (*P* = 0.05, *d* = 0.23, ITT population). The One Back test scores for subjects in the PQ912 group were slightly better (i.e., faster in response) compared to subjects in the placebo group. The mITT and PP analyses showed similar treatment effect sizes but these were not statistically significant. For the Detection test we found a treatment effect size of *d* = 0.21 (ITT population), but this was not significant. We found no PQ912 treatment effects on the composite scores of episodic memory, executive function, attention and overall cognition.

We found no differences in Mini Mental State Examination (MMSE) scores between the study groups.

Analysis of covariance (ANCOVA) analyses for cognitive endpoints identified no effects of gender, ApoE, country or age.

## Discussion

In this study we evaluated a novel therapeutic approach targeting the reduction of synaptotoxic and neurotoxic pGlu-Aβ via inhibition of the enzyme glutaminyl cyclase by treating 120 subjects with either PQ912 or placebo.

The purpose of the study was—by using a high dose of PQ912 resulting in a high QC occupancy—to optimize the chance to find both early-on safety and tolerability signs and any signal on various sensitive exploratory outcome measures in a relatively short time frame of 12 weeks to inform the design of a comprehensive phase 2b study. The selected outcome measures were tailored to the pathobiology of early AD with an emphasis on synaptic functional integrity (taking concurrently pharmacodynamic parameters related to the mechanism of action of PQ912 into account). Consequently, we introduced EEG recordings to capture potential effects on synaptic activity/integrity that directly relate to synaptic activity [19, 20]. In addition, level of neurogranin, a postsynaptic marker shown to be enhanced in AD [21], was measured. Both parameters are regarded as a centerpiece of pathology-related pharmacodynamic effects. As synaptic impairment directly results in cognitive memory deficits, the selection of sensitive cognitive tests in the NTB were used as efficacy readouts to test the hypothesis.

In a previously conducted large phase 1 study in healthy volunteers, PQ912 was considered safe and very well tolerated with no safety and tolerance signals identified. Therefore, the primary aim of this first in-patient study was to define the maximal tolerated dose of PQ912 and to identify the safety signal(s) associated with the MTD as proposed by the EMA guidelines for the development of new medicines for AD (Guideline on the clinical investigation of medicines for the treatment of Alzheimer’s disease, 22 February 2018 http://www.ema.europa.eu/docs/en_GB/document_library/Scientific_guideline/2018/02/WC500244609.pdf). To achieve this objective the highest multiple dose applied in the phase 1 study of 800 mg bid was used, achieving more than 90% QC inhibition in CSF [13]. The second aim was to evaluate exploratory but converging readouts for early signals of efficacy after the relatively short treatment period. Another aim of the study was to confirm the PK/PD correlation previously established in healthy volunteers, in the target population of early AD.

The safety and tolerability profile of 800 mg bid PQ912 as seen in this study indicate that the MTD has been reached. The results show an acceptable safety and tolerability profile with a comparable number of subjects reporting TEAEs or SAEs. However, more subjects treated with PQ912 discontinued treatment due to AEs specifically in the first 8 weeks of the exposure, with adverse events of the skin and subcutaneous organ system accounting for about half of the discontinuations. The types of skin adverse events were heterogeneous (reddening of skin/exanthema, rash, urticaria) and appeared 8–52 days after the start of treatment. There is currently no clear explanation for this observation, but similar skin reactions have been seen with other therapeutics (anti-epileptics, antibiotics, HIV drugs) suggesting that these events observed with PQ912 are caused by a hypersensitivity reaction to the drug or one of its metabolites; however, no data confirming such a hypothesis are currently available. As with other compounds inducing drug hypersensitivity, a slower titration schedule at the start of the treatment may allow for the adaptation of the immune system to the drug and reduce the number and severity of adverse events substantially. A slow titration regimen starting even with subpharmacological doses together with overall lower doses but still reaching a target occupancy on average of above 50% (see Fig. 6) should improve adherence to treatment in future studies. The higher incidence of gastrointestinal and skin and subcutaneous tissue disorder-related TEAEs in the PQ912 group compared to the incidence observed in the phase 1 study in healthy elderly subjects is probably due to a longer treatment duration (12 weeks vs 10 days). As in the latter phase 1 study, we confirmed a 90% TO in the patients in the current study, underlying the validity of the previously defined PK/PD model [13]. Of particular relevance is that there was no higher incidence or specific adverse event pattern observed in the central nervous system organ class in the PQ912 group compared to placebo, which could at least indirectly affect cognitive performance negatively.

The study also evaluated a potential treatment effect on selected physiological substrates that, in contrast to glutamate as in 3-Aβ, carry N-terminal glutamine, which is cyclized by QC as part of the maturation process of certain peptides and proteins. We chose the TRH and GnRH substrates as they have a high turnover, measured indirectly by quantifying the levels of TSH, T3 and T4 for the hypothalamic–pituitary–thyroid axis and testosterone for the hypothalamic–pituitary–gonadal axis. The finding that these parameters were not changed indicates also for patients that there is a high degree of on-target selectivity (glutamate vs glutamine), which has been shown in phase 1 and in animal safety studies [9, 13].

As regards exploratory biomarkers, the study revealed important pathology-related signals. There is now agreement that clinical pathological models of AD should be extended to include the effects of chronic inflammation. Consistent with this hypothesis [22, 23], YKL-40, a marker of astrocyte activation, has been shown to be increased in subjects with AD and MCI due to AD, compared to matched controls [21]. In this study, PQ912 significantly reduced YKL-40 by approximately 5% of baseline level, which was not observed in the placebo group within 12 weeks. The effect of QC inhibition might relate to pGlu-Aβ activating astrocytes in AD [24]. The absolute reduction is small but, in relation to the scope of increase reported to be 10% (in MCI due to AD) to 25% (in AD) compared to controls [25], the relative reduction amounts to 20–50% toward normal which is believed to be biologically meaningful.

The synaptic biomarker neurogranin was shown to be increased in AD compared to cognitively normal age-matched controls [21, 25]. Treatment with PQ912 reduced the neurogranin absolute level by approximately 4% in the PP population, although this decrease was not large enough to reach statistical significance. Similar to YKL-40, we consider the 4% decrease to be biologically meaningful considering that neurogranin is increased by about 35% at baseline in early AD disease versus healthy controls [25], thus in relative terms neurogranin was normalized by about 10% within the 3-month treatment period which might be very meaningful. Furthermore, a recent phase 3 study in prodromal AD observed that treatment with the Aβ antibody gantenerumab reduced CSF levels of neurogranin by about 10% after 104 weeks of treatment at the highest dose used and this change was classified as a clear indication of a reduction in synaptic dysfunction early in the disease [26]. The effects observed on YKL-40 and neurogranin levels are viewed as warranted to include these exploratory biomarkers in a future phase 2/POC study.

In the context of the concept of PQ912 inhibiting synaptic toxicity of pGlu-Aβ and an early AD population, EEG analysis may be particularly important as a sensitive index of synaptic impairment [19, 20] because an increase in relative theta power is regarded as the most sensitive oscillatory activity marker in the earliest stages of AD [27]. In the few previous EEG studies with approved symptomatic AD drugs, changes in spectral power measures toward normal values, including decreases in relative theta power, have been reported with rivastigmine and donepezil treatment [28]. In the current study, PQ912 treatment showed a significant positive effect on EEG oscillatory activity compared to placebo. More specifically, global relative theta power decreased in the PQ912 group, whereas it increased in the placebo group (i.e., consistent with disease progression). The significant difference in EEG theta power observed in this study with a small to moderate effect size is regarded as a pharmacodynamically important indicator and is considered a very suitable noninvasive biomarker for further proof-of-concept studies of PQ912. Importantly, each EEG recording was carefully monitored by qualified technicians in order to avoid drowsiness and artifacts, and central quality check procedures shortly following each EEG recording allowed a repeat recording when suboptimal data were obtained. Using these standardized procedures, we ascertained the use of artifact-free EEG data in the alert but relaxed state and an unlikely influence of these factors in the results. However, future studies are necessary to evaluate a true disease-modifying effect of PQ912 on synaptic function by demonstrating persistent differences in theta power after the cessation of the treatment period. Further, the analyses of the higher level functional connectivity using the phase lag index (PLI) and network topology measures, based on the minimum spanning tree of the full network, did not show differences between groups. As we would expect that changes in oscillatory activity, as reflected by relative power measures, are an expression of changes in functional connectivity between brain regions, we speculate that our selected measures were insensitive to the small effects of PQ912. The PLI and MST measures are highly ‘pure’ measures that were developed to exclude biases caused by volume conduction and differences in network sizes, respectively, but may have lost some relevant information due to these stringent calculation methods. We are now exploring the EEG dataset with other possibly more sensitive indices of functional connectivity [16, 29–31].

Analyses of RSfMRI parameters and use of eigenvector centrality mapping in clinical trials is in the experimental stage and there is no literature about regional differences in eigenvector centrality between groups of AD patients. The meaning of the statistically significant effect of PQ912 compared to placebo in the mean eigenvector centrality of the cuneus and lateral occipital regions (posterior) (effect size *d* = 0.58 observed for this difference) therefore remains unclear. Furthermore, in a previously published patient study, an increase in this parameter was observed in AD subjects compared to healthy controls [32]. A whole-brain analysis of voxelwise eigenvector centrality in the treated versus placebo groups was different compared to the AD patients in the previously published study, putting into question whether these characteristics of sampling locations can be used to measure treatment effect in AD. These regions did not have the same effect as in a study in AD subjects only. We therefore conclude that these sampling locations cannot be used to measure the treatment effect in AD.

**Fig. 5:**
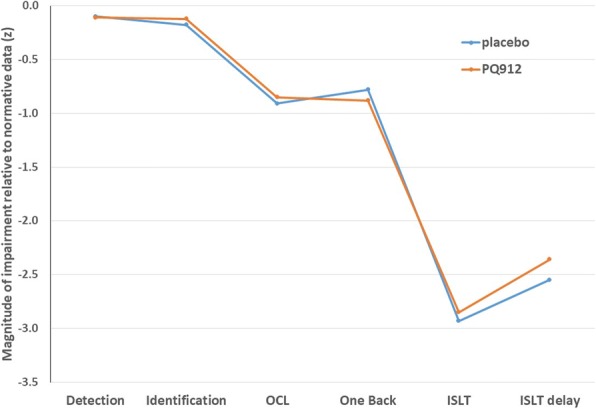
Magnitude of impairment on individual neuropsychological tests at baseline assessment (Cogstate). Impairment defined as group mean performance expressed standardized score (*z*) using age-matched normative data (mean and SD). ISLT International Shopping List Task, OCL One Card Learning

Although it was not expected to see clinically relevant changes in the complete NTB in a 12-week time period—expectation based on natural history cohorts and placebo arms of studies in a comparable early AD population (Cogstate normative data on file; Cogstate, New Haven, CT, USA)—we were interested to see whether the sensitive individual measures would move as an expression of synaptic recovery. In this context, it is noteworthy that an improvement in working memory was observed following treatment with PQ912. This improvement was accompanied by an improvement in alertness and, although moderate in magnitude, this treatment-related improvement was not large enough to reach statistical significance (effect size of both parameters: Cohen’s *d* > 00.2). Fig. 5 shows the degree of impairment at baseline of the placebo and PQ912 treatment groups for the individual cognitive tests when compared to Cogstate normative data for this early AD patient population (Cogstate normative data on file; Cogstate). The pattern of baseline impairment is as expected, with episodic memory being the most impaired area of function. The baseline deficit shown in the One Back Test, representing working memory, is a useful context for the treatment effect observed in this study (i.e., a 0.23 treatment effect in the context of a circa 0.7 deficit is a substantial return toward normal function). This might indicate early beneficial changes of PQ912 on cognition due to a more acute rescue effect on impaired synapses.

Regarding the exploratory readouts there are clear limitations in terms of treatment duration. One should note, however, that PQ912 has a new mechanism of action and one could not exclude it being reflected by a more acute effect on sensitive exploratory parameters as were selected for this study and which related to synaptic impairment. The results obtained are indeed giving important guidance for the design of the phase 2b study.

To test for efficacy as a disease modifier, as we intend to do in a phase 2b program, it is of course necessary to show significant effects on slopes of disease progression which would need a longer treatment duration in an adequately powered trial. The primary endpoint of the phase 2b program will be cognition. qEEG analysis will be incorporated as a secondary endpoint. As exploratory endpoints a set of biomarkers will be measured, including those biomarkers that showed a positive signal in this phase 2a study, such as neurogranin and YKL-40, and additionally a broader battery of CSF biomarkers (including synaptic proteins Contactin, Synaptosomal associated protein 25 and Growth associated protein 43; inflammatory markers sTREM2 and pGlu-CCL2; and markers of neurodegeneration NFL and VILIP1).

## Conclusions

In summary, the goal of this study was reached as key guidance for the design of a proof-of-concept study was obtained. Firstly, we could clearly establish a maximum tolerated dose of PQ912 and identify skin and subcutaneous tissue as well as gastrointestinal disorders as safety signals associated with a high dose. This is aligned with recommendation of the EMA guideline to establish the MTD in phase 1 or early phase 2. Secondly, the exploratory efficacy findings in this study are promising as the early signals found are all pointing in the same direction of improving function/inhibiting deterioration of synaptic activity and reducing neuroinflammation.

**Fig. 6:**
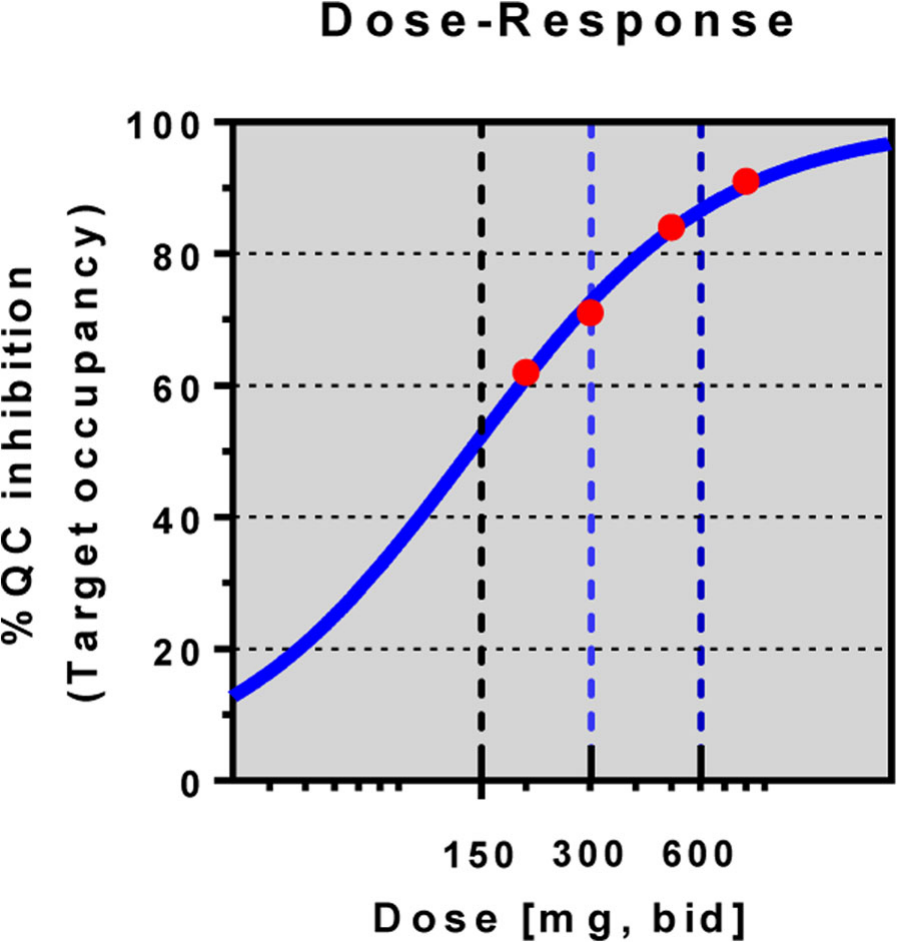
Dose–response curve for PQ912 with regard to target (QC enzyme) occupancy. bid twice daily, QC glutaminyl cyclase

The study also achieved its objective to guide development of PQ912 in future studies in early AD using a lower dose range of PQ912. Animal studies have shown that a TO of about 60% revealed a very robust effect in terms of improving special learning and memory deficits in AD-like mice in concert with significant reductions in pGlu-Aβ, and therefore a range of 50–70% of target occupancy should be evaluated in future studies to assess the efficacy of PQ912 in early AD. From data obtained in the phase 1 MAD study with PQ912 in elderly healthy volunteers [12] it can be derived that a 60% TO was achieved with a dose of 200 mg bid, thus with one quarter of the dose used in the SAPHIR study, and a dose of 300 mg bid will lead to 70% TO (Fig. 6) [13].

Lower doses and slower dose titration are highly likely to improve the safety and tolerance of PQ912—also because no relevant adverse events were seen in the first week of this study where all patients were on 400 mg bid. A lower dose would still have a high degree of QC inhibition and thus it is expected to increase the benefit–risk ratio. The therapeutic concept of QC inhibition has the potential to improve synaptic functioning by reducing synaptotoxic effects of pGlu-Aβ and long-term treatment may lead to a disease-modifying effect additionally supported by a reduction in neuroinflammation frequently associated with AD.

## Additional files

Additional file 1: Extended methods. (DOCX 144 kb)

Additional file 2: **Table S1.** Summary statistics and treatment effects of CSF, EEG, (RSf)MRI and cognition parameters. Difference (delta) plus 95% CI between treatment groups at EOT, controlled for baseline, age, gender, ApoE and country. Negative Cohen’s *D* indicates lower values at EOT in PQ912 group (less increase or more decrease). (PDF 89 kb)

## Abbreviations

AD: Alzheimer’s disease
AE: Adverse event
ANCOVA: Analysis of covariance
ApoE: Apolipoprotein E
Aβ: Amyloid beta
bid: Twice daily (bis in die)
CFT: Category Fluency Test
CI: Confidence interval
CSF: Cerebrospinal fluid
CTCAE: Common terminology criteria for adverse events
DMN: Default mode network
ECG: Electrocardiogram
EEG: Electroencephalography
EMA: European Medicines Agency
EOT: End of treatment
GnRH: Gonadotropin-releasing hormone
ISLT: International Shopping List Task
ITT: Intention to treat
*K*_i_: Inhibitory constant
LFT: Letter Fluency Test
MAD: Multiple ascending dose
MCI: Mild cognitive impairment
mITT: Modified intention to treat
MMSE: Mini Mental State Examination
MST: Minimum spanning tree
MTD: Maximum tolerated dose
NTB: Neuropsychological Test Battery
OCL: One Card Learning
PD/PK: Pharmacodynamics/pharmacokinetics
pGlu: Pyroglutamate
PLI: Phase lag index
PP: Per protocol
P-tau: Phosphorylated tau
QC: Glutaminyl cyclase
RD: Risk difference
RR: Relative risk
RSfMRI: Resting state functional magnetic resonance imaging
SAE: Serious adverse event
SD: Standard deviation
SOC: System organ class
T3: Triiodothyronine
T4: Prohormone thyroxine
TEAE: Treatment emergent adverse event
TO: Target occupancy
TRH: Thyrotropin-releasing hormone
TSH: Thyroid-stimulating hormone
YKL-40: Chitinase-3-like protein 1
