# Supplementary material for: Safety, tolerability and efficacy of the glutaminyl cyclase inhibitor PQ912 in Alzheimer’s disease: results of a randomized, double-blind, placebo-controlled phase 2a study
Source: Alzheimers Res Ther. 2018 Oct 12;10:107. doi: 10.1186/s13195-018-0431-6 (PMC6182869; doi:10.1186/s13195-018-0431-6)
Supplement: Supplementary file 1 — Extended methods. (DOCX 144 kb) [file 13195_2018_431_MOESM1_ESM.docx]

**Methods section extended**

**Study design**This was a multi-center, randomized, double-blind, placebo-controlled, parallel-group safety and tolerability Phase II study of PQ912. Efficacy was assessed in an exploratory manner.

Subjects satisfying all selection criteria at screening were randomly assigned in a 1:1 ratio to receive either PQ912 or placebo for 12 weeks. Dosage of PQ912 was 400 mg bid in the first week, followed by 800 mg bid for 11 weeks. During the study, visits to the study site occurred at baseline, after 3, 6 and 12 weeks on treatment and 28 days after the end of treatment.

All subjects who discontinued intake of study medication before 12 weeks of treatment were to continue with subsequent visits and undergo an EOT visit on the scheduled date of 12 weeks after baseline; a follow-up visit was only performed in case study medication was discontinued less than 28 days before Visit 5/EOT. Subjects completing the EOT assessments were considered completers of the study.

Randomization was done using a block size of 4, and stratification by site. The block-size and stratification rules were programmed by the EDC vendor in the EDC system that generated the random allocation sequence.

Each bottle containing study medication or placebo was labelled with a unique, randomly generated 6-digit kit number. At the time of randomization, allocation of treatment group and corresponding kit numbers was done by the EDC system. Participants were enrolled by the principal investigators of each site and his/her site staff. Blinding was double blind, so nor patient neither site staff knew the treatment arm the patient was allocated to. Sponsor and CRO staff involved in the day to day management of the trial was also blinded to study treatment.

Procedures at screening included documentation of medical history, physical and neurological examination, assessments of vital signs, ECG, EEG, MMSE, Geriatric Depression Scale, Neuropsychological Test Battery (two training sessions at separate visits) and MRI including resting state functional (RSf)MRI. Blood and urine samples were collected for blood chemistry, hematology, parameters related to QC substrates: thyroid stimulating hormone (TSH), total Triiodothyronine (T3), free Prohormone Thyroxine (T4), testosterone and for ApoE genotyping. A CSF sample was collected for biomarker assessments.

MMSE and Neuropsychological Test Battery were repeated at baseline and end of treatment (EOT); CSF sampling (within 24 hours after last dose), MRI, ECG and EEG were repeated at EOT only. Physical and neurological examination, vital signs, blood and urine sampling were repeated at each study visit. (S)AEs and concomitant medication were reported throughout the study.

Our Neuropsychological Test Battery included: “Paper and Pencil” tests: Letter Fluency test (LFT) and Category Fluency test (CFT) and computerized tests from the Cogstate battery (www.cogstate.com): International Shopping List test (ISLT), One Card Learning (OCL) test, One Back test, Detection test and Identification test.

At baseline and EOT, a 20-minute resting state EEG was recorded against a common reference at 21 electrode positions of the 10 – 20 system (Fp2 / Fp1, F8 / F7, F4/ F3, A2 / A1, T4 / T3, C4 / C3, T6 / T5, P4 / P3, O2 / O1, Fz, Cz, Pz). Sample frequency of these EEG recordings was ≥ 200 Hz. Filter settings were: high pass filter ≤ 0.2 Hz, low pass filter ≥ 70 Hz and no notch filter. Analog to digital conversion precision was ≥ 12 bit. Electrode impedance was kept below 5 kOhm. Patients were seated in a slightly reclined chair in a sound attenuated but fully lit room and were instructed to keep their eyes closed and stay awake. EEG technicians monitored the recording carefully and alerted the patients by sound stimuli at first signs of drowsiness. Five artefact free epochs per subject (± 8 seconds depending on sample frequency). EEG spectral analyses were performed with open-access software BrainWave (available at http://home.kpn.nl/stam7883/brainwave.html). Relative power in theta and alpha frequency bands (theta: 4 – 8 Hz, alpha: 8 – 13 Hz) and peak frequency (Hz; dominant frequency between 4 – 13 Hz) were calculated at each electrode using Fast Fourier Transformation. Values of the five epochs per subject were averaged to obtain values at subject level. Global relative theta and alpha power were calculated by averaging values of all 21 electrodes. Parietal-occipital peak frequency was calculated by averaging values of P4, P3, Pz, O2 and O1.

Central analysis/reading was done for clinical laboratory, genotyping, MRI images, EEG data and biomarkers. Cogstate performed central monitoring on the computerized test data.

**Prior and Concomitant medication**A maximum of 2 months of prior cumulative treatment with an acetylcholinesterase inhibitor or memantine was allowed if the acetylcholinesterase inhibitor or memantine was discontinued due to intolerance and if this was done at least 2 months prior to baseline. Use of Souvenaid was allowed if Souvenaid was discontinued at least 2 months prior to baseline, or if the subject was on stable dose for at least 6 months prior to baseline and was willing to continue during the study on the same dose and frequency. During the study period treatment with acetylcholinesterase inhibitor or memantine was allowed in case of clinically relevant worsening of cognitive performance. Treatment with anticoagulants, experimental medications or devices, immunosuppressive or chemotherapeutic agents, strong inhibitors or moderate inducers of the enzyme CYP2C19 or substrates with narrow therapeutic margin (fluconazole, fluvoxamin, ticlopidin, rifampicin, S-mephenytoin, repaglinide, phenytoin, phenobarbital and indomethacin), St. John’s Wort or any treatment which impaired cognitive function was not allowed during the study. For all other medications not intended to treat AD, the subjects had to be on a stable dose for at least 4 weeks prior to baseline. Medications which were central nervous system active and could affect cognitive function were not permitted during a period of 72 hours prior to cognitive testing. Hypnotics were not permitted during a period of 72 hours prior to EEG recording and had to be avoided during RSfMRI.

**Subjects**A total of 120 treatment-naïve, men (56) and women (64), aged 51-85 years, and with a diagnosis of mild cognitive impairment due to AD or mild dementia due to AD, according to the Alzheimer Association – National Institute on Aging criteria, were included in the study between March 2015 and December 2016. Other inclusion criteria were a Mini-Mental State Examination (MMSE) score between 21-30, and a brain magnetic resonance imaging (MRI) scan consistent with the diagnosis of the disease. In addition, subjects had to show one of the following: a) cerebrospinal fluid (CSF) sample with an Aß 1-42 concentration < 638 ng/L AND total tau > 375 ng/L; b) CSF sample with an Aß 1-42 concentration < 638 ng/L AND p-tau > 52 ng/L; c) Tau/Aß ratio > 0.52; or d) Positive amyloid positron emission tomography if availablea positive AD biomarker signature at screening. Subjects with neurologic diseases other than AD, with atypical clinical presentation of mild cognitive impairment due to AD or mild dementia due to AD (such as the visual or language variant of AD), with major psychiatric disorders (such as major depressive disorder), or with other clinically important diseases or conditions that could compromise the study or their safety, were excluded from the study. Women were surgically sterile or postmenopausal.

**Safety and Tolerability endpoints**
Two primary safety and tolerability composite endpoints were defined: Safety, based on: Discontinuation of subject due to serious adverse event (SAE), or Discontinuation of subject due to adverse event (AE) with severity ≥ 3 according to Common Terminology Criteria for Adverse Events (CTCAE), or Discontinuation of subject due to an extreme laboratory parameter. Tolerability, based on: Dose adjustment during treatment period, and/or Non-adherence to randomized treatment.

An SAE was defined as any untoward medical occurrence that at any dose resulted in death; was life threatening; required inpatient hospitalization or prolongation of existing hospitalization; resulted in persistent or significant disability/incapacity (with disability defined as a substantial disruption of a person’s ability to conduct normal life functions); or was a congenital anomaly/birth defect. Important medical events may have been considered a serious adverse drug experience when, based upon appropriate medical judgment, they could have jeopardized the subject and required medical or surgical intervention to prevent one of the outcomes listed above.

The verbatim term of each AE was mapped to a System Organ Class (SOC) and Preferred Term (PT) using the Medical Dictionary for Regulatory Activities (MedDRA) Dictionary.

Dose adjustment was defined as a reduction of dose from 800 mg bid to 400 mg bid. Non-adherence was defined as using <75% of the prescribed dose in 4 consecutive weeks including at least 1 week with <80%, or ≥3 consecutive days in total or 7 days of interrupted use during the full 12 weeks.

Secondary tolerability endpoints were: number of SAEs and AEs with severity ≥ 3 according to CTCAE, the time to first appearance of AEs, time to dose adjustment, time to non-adherence, and a composite of time to dose adjustment, non­adherence, discontinuation due to SAE, AE with severity ≥ 3 according to CTCAE or extreme laboratory parameters.

Other safety measures were: AEs, vital signs, ECG measurements, clinical laboratory tests, changes on brain MRI scans, physical and neurologic examinations.

**Efficacy endpoints**
The following endpoints were defined for exploratory efficacy: CSF biomarker endpoints: Diagnostic biomarkers: Aß 1-42; Tau; P-tau (Innotest, Fujirebio) Exploratory biomarkers: QC activity, primary target of inhibitor (Evotec AG); Neurogranin and Beta secretase I (ADxNeurosciences/Euroimmun); Contactin 2 (R and D systems, duoset); Neurofilament light chain (Uman diagnostics); Chitinase-3-like protein 1 (CHI3L1 = YKL-40) (Quidel Corporation).

Neuronal oscillatory activity and network endpoints: EEG endpoints: mean peak frequency in the parieto-occipital region; global relative alpha and theta power (8 – 13 Hz and 4 – 8 Hz, respectively); mean global Phase Lag Index in the alpha band; network topology measures in the alpha band, based on the minimum spanning tree of the full network: mean Phase Lag Index, leaf fraction, and tree hierarchy in the alpha band.

MRI endpoints: Normalized brain volume at screening and Percentage brain volume change at EOT

RSfMRI endpoints: Mean z-statistic default mode network (DMN); Mean Eigenvector centrality values and Mean path length and clustering coefficient

Cognitive endpoints: Episodic Memory (average of standardized scores of the OCL Test, ISLT and ISLT-delayed recall); Executive function (average of standardized scores of LFT, CFT and One Back Test); Attention (average of standardized scores of Detection Test and Identification Test.); Overall Cognition (average of standardized scores of all cognitive measures. Scores of at least 6 tests needed for calculation) MMSE

**QC activity and inhibition**

QC activity in CSF was measured by Evotec AG using a semi-automated fluorimetric assay as described in Lues et al. in detail. Briefly, the substrate Gln-AMC is cyclized by QC to pGlu-AMC. The fluorophore AMC is subsequently released by the auxiliary enzyme pyroglutamyl aminopeptidase and measured at an Extinction/Emission of 380/460 nm. For each assay 1 part of CSF sample and 2 parts of reagents (Substrate and auxiliary enzyme in assay buffer) were used. It has to be mentioned that this dilution of CSF in the assay leads also to a dilution of the competitive inhibitor PQ912 in the assay. Therefore QC activity in inhibitor containing samples is generally overestimated compared to the undiluted conditions in vivo.

The in vivo QC target occupancy (TO) was calculated from PQ912 CSF levels for CSF samples collected within 24 hours after last compound intake at EOT visit. TO was calculated using the following formula: TO (%) = 100*C/(Ki+C). Where are: TO = target occupancy in %, Ki = Inhibitory constant of PQ912 = 25 nM and C = measured CSF concentration of PQ912.

PQ912 levels in CSF were determined using a validated liquid chromatography with tandem mass spectrometric detection method by Swiss BioQuant which includes protein precipitation and on-line solid-phase extraction as sample preparation steps.
