# Supplementary material for: Safety, tolerability and efficacy of the glutaminyl cyclase inhibitor PQ912 in Alzheimer’s disease: results of a randomized, double-blind, placebo-controlled phase 2a study
Source: Alzheimers Res Ther. 2018 Oct 12;10:107. doi: 10.1186/s13195-018-0431-6 (PMC6182869; doi:10.1186/s13195-018-0431-6)
Supplement: Supplementary file 2 — Table S1. Summary statistics and treatment effects of CSF, EEG, (RSf)MRI and cognition parameters. Difference (delta) plus 95% CI between treatment groups at EOT, controlled for baseline, age, gender, ApoE and country. Negative Cohen’s D indicates lower values at EOT in PQ912 group (less increase or more decrease). (PDF 89 kb) [file 13195_2018_431_MOESM2_ESM.pdf]

Table S1: Summary statistics and treatment effects of CSF, EEG, (RSf)MRI and cognition parameters. Difference (delta) plus 95% Confidence Intervals between treatment groups at EOT , controlled for baseline, age, gender, ApoE and country is presented . Negative Cohen's D indicate lower values at EOT in PQ912 group (less increase or more decrease).

CSF: median (Q1 - Q3) for baseline and EOT for groups placebo and PQ912, ANCOVA analyses on PP dataset and on log10-transformed values

parameter	placebo				PQ912				results ANCOVA			
	baseline		EOT		baseline		EOT		N	delta (95%CI)	p	Cohen's D
	N	median (Q1 - Q3)	N	median (Q1 - Q3)	N	median (Q1 - Q3)	N	median (Q1 - Q3)				
Total-Tau in CSF (pg/mL)	42	710 (485 - 974)	42	620.5 (511 - 947)	26	651 (514 - 934)	26	678.5 (485 - 874)	66	-0.01 (-0.038 ; 0.018)	0.485	-0.05
Amyloid-Beta in CSF (pg/mL)	42	549 (493 - 617)	42	559.5 (483 - 647)	26	517.5 (492 - 567)	26	538.5 (477 - 578)	66	-0.002 (-0.034 ; 0.03)	0.896	-0.02
Total-Tau/Amyloid-Beta in CSF	42	1.315 (0.931 - 1.734)	42	1.162 (0.863 - 1.890)	26	1.311 (0.931 - 1.824)	26	1.232 (0.841 - 1.668)	66	-0.026 (-0.066 ; 0.013)	0.187	-0.12
Phospho-Tau in CSF (pg/mL)	42	87.5 (72 - 117)	42	86 (72 - 114)	26	86 (69 - 106)	26	86 (66 - 114)	66	-0.001 (-0.023 ; 0.022)	0.951	-0.01
Glutaminyl cyclase activity (mU/L)	41	120.8 (101.3 - 140.2)	41	120.3 (104.1 - 137.5)	26	110.2 (94.8 - 134.2)	26	35.6 (29.7 - 63.2)	65	-0.454 (-0.507 ; -0.4)	<.001	-1.66
Neurogranin (pg/ml)	41	419 (348 - 583)	41	411 (328 - 517)	26	422 (314 - 530)	26	391 (298 - 516)	64	-0.021 (-0.054 ; 0.011)	0.194	-0.12
BACE1 (pg/ml)	41	2154 (1847 - 2525)	41	2165 (1798 - 2554)	26	2061.5 (1767 - 2388)	26	1928 (1758 - 2470)	64	0.002 (-0.012 ; 0.016)	0.788	0.02
Contactin2 (ng/ml)	41	72.98 (54.75 - 83.8)	41	69.86 (52.38 - 86.95)	26	64.08 (54.77 - 77.56)	26	63 (51.37 - 85.43)	64	-0.006 (-0.049 ; 0.037)	0.789	-0.03
NFL (pg/ml)	41	1164 (839 - 1491)	41	1230 (888 - 1493)	26	1186 (921 - 1800)	26	1185 (930 - 1632)	64	0.035 (-0.031 ; 0.101)	0.290	0.18
YKL40 (pg/ml)	41	367 (295 - 476)	41	353 (274 - 469)	26	292 (232 - 412)	26	297 (219 - 384)	64	-0.032 (-0.059 ; -0.004)	0.025	-0.20
Ng/BACE1	41	0.206 (0.174 - 0.24)	41	0.205 (0.171 - 0.226)	26	0.201 (0.160 - 0.228)	26	0.193 (0.163 - 0.215)	64	-0.019 (-0.044 ; 0.005)	0.114	-0.16
P-Tau/T-Tau	42	0.134 (0.119 - 0.144)	42	0.127 (0.118 - 0.147)	26	0.122 (0.112 - 0.138)	26	0.13 (0.111 - 0.139)	66	-0.002 (-0.029 ; 0.026)	0.909	-0.02
pEAbeta oligomer	42	0.01 (0.00 - 0.54)	42	0.26 (0.00 - 0.58)	25	0.51 (0.22 - 0.99)	26	0.44 (0.00 - 1.42)		n.a.		
pEAbeta monomer	42	0.8 (0.00 - 1.33)	42	0.67 (0.01 - 1.85)	25	0.98 (0.11 - 2.18)	26	1.1 (0.40 - 2.11)		n.a.		

EEG: mean (SD) for baseline and EOT for groups placebo and PQ912, ANCOVA on imputed ITT dataset

parameter	placebo				PQ912				results ANCOVA			
	baseline		EOT		baseline		EOT		N	delta (95%CI)	p	Cohen's D
	N	mean (SD)	N	mean (SD)	N	mean (SD)	N	mean (SD)				
MEAN PEAK FREQUENCY PARIETO-OCCIPITAL (Hz) AVG, 1st level	60	8.8664 (1.2332)	55	8.7188 (1.1766)	60	8.8611 (1.3633)	47	8.7294 (1.3994)	100	-0.017 (-0.435 ; 0.402)	0.938	-0.01
Log10-transformed ALPHA RELATIVE POWER GLOBAL AVG, 1st level	60	0.2924 (0.1509)	55	0.2859 (0.1316)	60	0.2778 (0.1376)	47	0.2747 (0.137)	100	0.008 (-0.038 ; 0.053)	0.746	0.04
Log10-transformed THETA RELATIVE POWER GLOBAL AVG, 1st level	60	0.1647 (0.0794)	55	0.1762 (0.0819)	60	0.1763 (0.0976)	47	0.1695 (0.0881)	100	-0.057 (-0.093 ; -0.021)	0.002	-0.29
THETA / ALPHA RELATIVE POWER GLOBAL, 1st level	60	0.788 (0.683)	55	0.786 (0.597)	60	0.847 (0.850)	47	0.840 (0.728)	100	-0.026 (-0.126 ; 0.073)	0.599	-0.04
ALPHA PLI GLOBAL AVG, 2nd level, functional connectivity	60	0.1837 (0.0702)	55	0.1884 (0.0765)	60	0.1767 (0.0634)	47	0.1737 (0.0652)	100	-0.003 (-0.021 ; 0.015)	0.778	-0.04
ALPHA MST LEAF FRACTION GLOBAL AVG, 3rd level	60	0.5815 (0.0565)	55	0.5884 (0.0613)	60	0.5868 (0.0473)	47	0.5851 (0.0459)	100	-0.007 (-0.027 ; 0.013)	0.475	-0.13
ALPHA PLI MEAN OF MST AVG, 3rd level	60	0.3866 (0.1076)	55	0.3932 (0.1185)	60	0.3754 (0.1007)	47	0.3676 (0.0969)	100	-0.006 (-0.031 ; 0.019)	0.620	-0.06
ALPHA MST TREE HIERARCHY GLOBAL AVG, 3rd level	60	0.3955 (0.032)	55	0.3995 (0.0323)	60	0.4022 (0.0267)	47	0.4033 (0.0319)	100	-0.002 (-0.015 ; 0.011)	0.751	-0.06

RSfMRI: mean (SD) for baseline and EOT for groups placebo and PQ912, ANCOVA on imputed ITT dataset

parameter	placebo				PQ912				results ANCOVA			
	baseline		EOT		baseline		EOT		N	delta (95%CI)	p	Cohen's D
	N	mean (SD)	N	mean (SD)	N	mean (SD)	N	mean (SD)				
PERCENTAGE BRAIN VOLUME CHANGE (basic MRI)	59	1406.7 (94.9)	59	change= -0.3 (0.6)	60	1386 (80)	54	change= -0.6 (0.8)	109	-0.143 (-0.41 ; 0.124)	0.291	-0.20
Log10-transformed MEAN Z-STATISTICS DEFAULT MODE NETWORK	59	6.15 (1.84)	47	5.59 (2.35)	60	6.16 (1.93)	41	4.99 (1.68)	86	-0.019 (-0.082 ; 0.045)	0.560	-0.11
MEC CUNEUS AND LATERAL OCCIPITAL REGIONS*	59	6.11 (0.10)	47	6.14 (0.10)	60	6.09 (0.12)	41	6.08 (0.11)	86	-0.062 (-0.105 ; -0.02)	0.005	-0.58
MEC ANTERIOR CINGULATE AND PARACINGULATE GYRUS*	59	6.00 (0.13)	47	5.98 (0.13)	60	6.02 (0.12)	41	5.99 (0.09)	86	-0.004 (-0.051 ; 0.044)	0.880	-0.03
MEAN CLUSTERING COEFFICIENT*	59	2.43 (0.68)	47	2.52 (0.73)	60	2.53 (0.73)	41	2.49 (0.83)	86	0.037 (-0.272 ; 0.346)	0.812	0.05
MEAN PATH LENGTH*	59	22.96 (2.95)	47	23.72 (3.63)	60	23.14 (4.45)	41	22.89 (4.09)	86	-0.963 (-2.766 ; 0.84)	0.291	-0.25

Cognition: mean (SD) for baseline and EOT for groups placebo and PQ912, ANCOVA on imputed ITT dataset

parameter	placebo				PQ912				results ANCOVA			
	baseline		EOT		baseline		EOT		N	delta (95%CI)	p	Cohen's D
	N	mean (SD)	N	mean (SD)	N	mean (SD)	N	mean (SD)				
Episodic Memory (standardised AVG(OCL,ISRL,ISL))	60	-0.045 (0.766)	60	-0.017 (0.862)	60	0.045 (0.783)	52	0.076 (0.835)	109	-0.113 (-0.31 ; 0.084)	0.259	-0.13
Executive Function (standardised AVG(LFT,CFT,ONB))	60	-0.011 (0.676)	60	-0.035 (0.675)	60	0.011 (0.707)	52	0.046 (0.882)	109	0.009 (-0.155 ; 0.173)	0.911	0.01
Attention (standardised AVG(DET,IDN))	60	0.015 (0.806)	60	-0.154 (0.865)	60	-0.015 (0.866)	52	-0.067 (1.02)	109	0.117 (-0.162 ; 0.396)	0.406	0.13
Overall Cognition (standardised AVG(all cognitive measures))	60	-0.017 (0.564)	60	-0.058 (0.634)	60	0.017 (0.563)	52	0.029 (0.696)	109	-0.02 (-0.141 ; 0.1)	0.738	-0.03
MMSE score	60	24.8 (3.3)	60	25 (3.4)	60	25.2 (3.0)	55	24.6 (3.0)	112	-0.466 (-1.269 ; 0.337)	0.252	-0.15
**subtests**												
LFT - Number of words-Total	60	35.9 (12.2)	60	36.1 (12.2)	60	37 (11.3)	54	36 (13.6)	111	-1.847 (-4.817 ; 1.122)	0.220	-0.14
CFT - Number of words-Total	60	13.8 (5.4)	60	13.9 (5.6)	60	14.1 (4.9)	54	13.9 (6.1)	111	-0.405 (-1.947 ; 1.137)	0.603	-0.07
Detection (log10 ms)	60	2.569 (0.143)	60	2.592 (0.127)	60	2.568 (0.137)	52	2.565 (0.151)	109	-0.029 (-0.075 ; 0.017)	0.221	-0.21
Identification (log10 ms)	60	2.734 (0.067)	60	2.747 (0.078)	60	2.739 (0.081)	52	2.749 (0.087)	109	-0.003 (-0.026 ; 0.02)	0.795	-0.04
One-card learning (arcsine square root)	60	0.859 (0.103)	60	0.868 (0.105)	60	0.865 (0.085)	52	0.866 (0.103)	109	-0.016 (-0.05 ; 0.019)	0.372	-0.15
One-back memory (log10 ms)	60	3.018 (0.130)	60	3.028 (0.128)	60	3.028 (0.120)	52	3.011 (0.129)	109	-0.03 (-0.06 ; 0)	0.050	-0.23
International Shopping List - Summary of all trials	60	14.5 (4.5)	60	14.7 (4.9)	60	14.8 (4.6)	52	15.2 (5.1)	109	-0.481 (-1.888 ; 0.927)	0.499	-0.10
International Shopping List - Recall	60	3.0 (2.6)	60	2.8 (2.6)	60	3.4 (2.7)	52	3.3 (2.7)	109	-0.011 (-0.694 ; 0.672)	0.975	0.00

* values are multiplied by 1000
MEC = Mean Eigenvector Centrality
